# A System of Practical Medicine

**Published:** 1885-07

**Authors:** 


### Book Reviews.

---

A System of Practical Medicine by American Authors. Edited by William Pepper, M. D., L.L. D., Provost and Professor of the Theory and Practice of Medicine and of Clinic Medicine in the University of Pennsylvania. Assisted by Louis Starr, M. D., Clinical Professor of Diseases of Children in the Hospital of the University of Pennsylvania. Vol. II, General Diseases (continued) and Diseases of the Digestive System. Philadelphia: Lea Brothers & Co. 1885.

This second volume, containing 1312 pages of matter, which is eminently appropriate after the articles of the first volume, exhibits the highly practical character of the work.

The manner in which the various subjects are treated by the several authors serves further to recommend the thoroughness and exactness of their investigations. While the plan of the undertaking is not perhaps that which is best suited to a book of reference for the busy practitioner who desires to consult authorities upon special diseases, yet the division of general and reginal disorders has its advantages for the student of the derangements of the various functions. The first topic being rheumatism, by R. P. Howard, M. D., is generalized under the heads of acute, sub-acute and chronic, without, however, grasping the cardinal doctrine of fibrinitis, which is undoubtedly the underlying element of the rheumatic diathesis; neither does he give that prominence to the disturbance of the chylopoetic viscera dependent upon hepatic implication, which has been recognized as an essential feature of this disease. Great prominence is given to the consideration of the salicylates in the treatment, with constitutional alteratives, to the neglect of the mercurial influence that is now regarded by many as the most efficacious agent in combating the cardiac troubles which are so important to be promptly arrested. While great research is exhibited in the presentation of the views of others as to the protean forms of this affection in the different tissues of the organism, we are left to infer that palliative rather than radical means of correcting the cause of trouble are only available in its treatment.

Gout, by W. H. Draper, M. D., is attributed to advanced civilization, and is alike the product of the luxury and the misery which a high civilization entails. In the evolution of the disease the joints, where the connective tissue is most dense and the least vascular, suffer earliest ; at a later period the connective tissue of the blood vessels, nerves and viscera becomes subject to gouty changes. Gradually the dyscrasia becomes more profound, and the constitutional symptoms and structural changes which belong to the atonic and irregular forms of the disease are developed. It is noteworthy that the constitutional symptoms are not as severe as in rheumatism. As the author considers that none of the theories of the production of the lithæmic state harmonize all its phenomena, only general indications for correcting it are presented as the aims of the treatment of the vice upon which gout depends.

Rachitis, by Abraham Jacobi, M.D., presents defective calcification of the forming bone as one of its principal characteristics, and it has its predisposition and its direct and proximate causes. It is in the period of rapid development that rachitis is observed. According to our author's views, only a certain amount of phosphate of lime is useful in rachitis and in digestive disturbances ; but his experience with phosphorus in other diseases of the bones encourages the belief that it will accomplish much in the treatment of rachitis. The meat-soups, cod-liver oil, regimen, and hygienic management are chiefly relied upon as preventive and curative measures.

Scurvy, by Philip S. Wales, M. D., arises, in his view, from prolonged employment of food deficient in succulent or fresh vegetable matter, and progresses uniformly to a fatal issue, in a longer or shorter time, if the dietetic errors remain uncorrected. The first object should be to supply the patient with lemon juice and fresh vegetables. Should, however, convalescence be delayed, the vegetable bitters with mineral acids and ferruginous tonics and quinia will furnish useful adjuncts. Mouth washes composed of solutions of chlorated lime, potassium permanganate, carbolic acid, etc., are recommended, and we would add, as another, the popular Myrrh Mixture. Should ulceration attack the legs, as is often the case, mild astringents and stimulating applications with attention to cleanliness are indicated, and perhaps nothing serves a better purpose than a weak solution of sulphuric acid with lint to the sores.

Purpura, by I. E. Atkinson, M. D., occupies a small space under the three forms of purpura simplex, purpura hæmorrhagica and purpura rheumatica. In the treatment, we are told, and accredit, that the tincture of the chloride of iron is the best remedy.

Diabetes Mellitus, by James Tyson, M. D., comes in for a full share of attention as a grave and not unfrequent disorder. It is associated with a derangement of the sugar-assimilating office of the liver, as the result of which an abnormally large quantity of glucose is poured into the hepatic veins and thence into the systemic blood, from which it is secreted by the kidneys.

Discrediting the investigations of Dickinson, on the alterations in the nerve centers, brain and spinal column, our author lays more stress upon the changes in the pancreas and liver as bearing upon diabetes, while the kidneys become involved only secondarily, as a consequence of over-work in the copious secretion of urine. Unless it be that the indigo test prove more delicate, our author considers that form of cupric test, known as Fehling's solution, the most satisfactory for general use in testing the urine for sugar.

Preference is given to the skim-milk treatment of diabetes, and in case of failure with the dietetic management of it, the codiene may be given in doses of ¼ grain three times a day, increasing by ¼ grain daily until the sugar disappears, or the remedy ceases to have any effect, or until drowsiness ensues.

Ergot is held to be next in efficacy. Bromide of potash may be useful in certain cases of nervous origin. The various preparations of phosphates are useful. Potassium carbonate is a type of the alkaline treatment, which has been given in combination with the aromaitc spirits of ammonia. Different other medicines have been attended with variable results.

Scrofula, by John S. Lynch, M. D., is treated upon the basis of two types—the torpid and sanguine—and iodine holds the chief place as a remedy.

Heriditary Syphilis, by William White, M. D., must be read to be appreciated properly.

Diseases of the Mouth and Tongue, by Sol. Cohen, M. D., occupy very properly a place under the heading of diseases of the digestive system, followed by diseases of the tonsils, diseases of the pharynx, and diseases of he œsophagus, by the same author, filling 111 pages of the book.

Functional and Inflammatory Diseases of the Stomach, by Samuel G. Armor, M. D., LL. D., takes quite a different view of diet from that of Dr. Flint, Sr., and instead of leaving the patient to his inclinations claims that the physician should most carefully select the patient's food and urgently insist on its exclusive use.

Simple Ulcer of the Stomach, by W. H. Welch, M. D., cancer of the stomach, hemorrhage from the stomach, dilatation of the stomach, and minor organic affections of the stomach, by the same author, take up 139 pages, which would make a considerable volume of the style of the Birmingham Library.

Intestinal indigestion, constipation, euterolgia (intestinal colic), acute intestinal catarrh (duodemitis, jejunitis, ileitis, colitis, proctitis), chronic intestinal catarrh and cholera-morbus, all by W. W. Johnston, M. D., fill 105 pages. Among many problematical views in these papers, the advocacy of opium as a curative agency *per se* may perhaps be questioned by some, yet it seems founded upon correct principles in view of spasm and pain in acute intestinal catarrh.

Intestinal Affections of Children in Hot Weather, by J. Lewis Smith, M. D., which tell so largely in the death statistics in our southern country, are treated without calomel, and no reference is made to the time-honored hydrag., cum creta, thus failing to rectify the hepatic trouble in these disorders.

Pseudo-Membranous Enteritis, by Philip S. Wales, M. D., affords rather an unpromising outlook for medication, and depends much upon regimen and diet for a cure.

Dysentery, typhlitis, perityphlitis and peratyphlitis, intestinal ulcer, and hemorrhage from the bowels, by James T. Whitaker, M. D., make up 59 pages among the most important of this volume, from the frequency of the cases, and the imperfect means of diagnosing the hidden inflammations of the intestines.

Intestinal obstruction, by Hunter McGuire, M. D., manifests a decided tendency to *masterly inactivity* in cases not demanding surgical interference, and he takes exception to pound doses of quicksilver and to the internal development of gas by chemical decomposition of gas by chemical decomposition.

Cancer and Lardaceous Degeneration of the Intestines, by I. E. Atkinson, depend upon causes that only can be reached through the general system.

Diseases of the Rectum and Anus, by Thomas G. Morton, M. D., and Henry G. Wetherell, M. D., Ph. G., embodies such a variety of troubles that no passing notice can give an idea of its scope, yet it strikes us that few will concur in confining nutrient injections to the rectum when the large mucous surface of the colon above the sigmoid flexure may be made subservient to this end by the use of a long elastic tube to the syringe.

Intestine Worms, by Joseph Leidy, M. D., sums up the most essential points connected with eutozoa in 35 pages, and gives the preference to oil of turpentine for expelling tape worm.

Diseases of the Liver, by Roberts Bartholow, A. M., M. D., LL. D., should be read and carefully studied by all into whose hands this work is placed, being an epitome of the present knowledge on this subject in 146 pages.

Diseases of the Pancreas, by Louis Starr, M. D., is highly instructive.

Peritonitis, by Alonzo Clark, M. D., LL. D., is pre-eminently practical and timely.

Diseases of the Abdominal Glands (Tabes Messenterica), by Samuel C. Busey, M. D., claims that faulty nutrition is the predominant factor, and the drugs employed should be directed to the improvement of the assimilative functions.

Such a summary of contents cannot fail to impress all with the value of this important contribution to medical progress.

